# Optimising the Use of Staging Computed Tomography of the Chest, Abdomen and Pelvis (CT CAP) in Newly Diagnosed Brain Lesions in Adults

**DOI:** 10.7759/cureus.114561

**Published:** 2026-08-15

**Authors:** Ameerah Ilyas

**Affiliations:** 1 Ophthalmology, Royal Victoria Infirmary, Newcastle, GBR

**Keywords:** ct scan head, ct staging, diagnostic delay, solitary brain lesions, treatment protocols

## Abstract

Background

Newly diagnosed brain lesions (NBLs) present a diagnostic challenge in distinguishing primary from secondary (metastatic) disease. Staging CT of the chest, abdomen and pelvis (CT CAP) is frequently used to identify an extracranial primary; however, in the absence of clear selection protocols, this investigation is often applied indiscriminately, exposing patients with primary brain tumours to unnecessary radiation, cost and delay. This study aimed to identify CT head-based predictors of a positive CT CAP to inform a selective staging protocol.

Methods

This retrospective cohort study included 184 patients reviewed at the Neurosurgery multidisciplinary team (MDT) at Queen Elizabeth Hospital, Birmingham (April 2022 to November 2022) with an NBL who underwent CT head imaging. Candidate predictors - lesion location (supratentorial/infratentorial), lesion number, lesion size, and previous history of cancer - were assessed against CT CAP outcome using univariable and multivariable logistic regression.

Results

A definitive CT CAP outcome was available for 106 patients, of whom 19 (18%; 95% confidence interval (CI), 11-27%) had a positive result. On univariable analysis, previous cancer history (odds ratio (OR), 17.8; 95% CI, 5.17-61.0), lesion size ≥4 cm (OR, 3.73; 95% CI, 1.01-13.8), and increasing lesion number (OR, 7.33 for ≥3 lesions; 95% CI, 1.94-27.7) were associated with a positive result, whereas lesion location was not (P = 0.50). After multivariable adjustment, only previous cancer history remained independently associated with a positive CT CAP (adjusted OR, 14.6; 95% CI, 4.09-52.4; P < 0.001). Previous history of cancer is the dominant independent predictor of a positive staging CT CAP in patients with NBLs.

Conclusion

Previous history of cancer is the sole independent predictor of a positive staging CT CAP in patients with an LBN, whereas lesion number, size, and location are not. A risk-stratified staging pathway that prioritises cancer history rather than applying CT CAP indiscriminately could reduce unnecessary imaging while preserving diagnostic yield for metastatic disease.

## Introduction

Newly diagnosed brain lesions (NBLs) can often pose a significant diagnostic challenge due to the subsequent investigations that arise to determine if the lesion is a primary brain lesion or metastatic [1]. For patients with secondary brain lesions, a staging CT chest, abdomen, pelvis (CT CAP) is often used to identify the primary malignancy and extent of the extracranial disease [2]. However, in the absence of clear protocols, the current practice can lead to all patients with NBLs receiving a staging CT CAP, regardless of whether the lesion is primary or secondary. This can result in a significant proportion of patients with primary brain lesions undergoing unnecessary investigations. The consequences of indiscriminate use of CT CAP include unnecessary radiation exposure with an effective dose of approximately 20-25 mSv to the patient [3,4], in addition to the financial costs, consumption of valuable healthcare resources, and delays in patient management. Numerous protocols have been developed to guide CT imaging, including the use of CT imaging in minor head injuries [5] and selection of CT chests in blunt trauma, which have effectively reduced unnecessary CT examinations by 25% [6]. However, there have been no equivalent protocols developed for the selective use of CT CAP in patients with NBLs. Previous studies have suggested protocol variables that could be implemented to provide a structured approach to the use of CT CAPs in diagnoses [7].

The frequency of primary brain tumours is 7.25 per 100,000, and in the United Kingdom alone, 9,000 patients are diagnosed annually with primary brain tumours. Hence, there is a significant diagnostic burden on patients with NBLS [8]. Creating a set of variables based on CT head characteristics, which can differentiate primary from secondary brain lesions, could allow for a selection criterion for patients who may require subsequent staging CT CAP. Recognised radiological features may help differentiate metastasis from primary brain lesions, including multiple lesions, lesion size, anatomical location, with infratentorial lesions more suspicious for metastases, and the known history of extracranial malignancy [8,9]. This study aims to identify predictors of secondary brain lesions based upon CT head findings, to develop a clinical protocol that would guide the selection of patients for CT CAP staging. Creating evidence-based criteria for when a CT CAP is indicated can reduce unnecessary investigations, minimise patient exposure to ionising radiation and decrease the financial and time costs associated with unnecessary investigations while maintaining diagnostic sensitivity for metastatic disease.

## Materials and methods

Study design

This was a retrospective cohort study at a tertiary hospital in the multidisciplinary team (MDT) neurosurgery at Queen Victoria Hospital. It included 184 patients who were reviewed in the Neurosurgery MDT meetings and underwent diagnostic evaluation for NBLs from April 2022 to November 2022. This study has received institutional approval as a Quality Improvement Project. The study adhered to the tenets of the Declaration of Helsinki. Two independent investigators assessed the cranial imaging and associated factors. Clinical data were obtained from a review of case notes, including age, gender, type of cranial imaging, location of lesion, size of lesion, comorbidities, and results of CT CAP.

Data collection

Data were extracted retrospectively from electronic case notes and radiology reports for all patients reviewed at the Neurosurgery MDT. Two independent investigators reviewed the CT head and CT CAP reports to record lesion characteristics and staging outcome; discrepancies were resolved by consensus. Variables with incomplete documentation were recorded as missing rather than imputed.

Inclusion Criteria

Patients reviewed at the Neurosurgery MDT between April 2022 and November 2022 with an NBL, who had undergone an initial CT head followed by subsequent staging CT CAP, were included.

Exclusion Criteria

Patients were excluded if they did not undergo CT CAP, or if they had a known pre-existing diagnosis of cancer with established metastatic disease at the time of brain lesion detection. This exclusion was applied to focus the cohort on patients in whom CT CAP was being used as a genuine diagnostic or staging investigation for a newly identified lesion, rather than as surveillance imaging in a setting where metastatic disease had already been confirmed.

Candidate Variables

Candidate predictor variables recorded for each patient included age, gender, date of referral, lesion number, lesion size, lesion location, and previous history of cancer (recorded separately from other comorbidities, given its established relevance to pre-test probability of metastatic disease). The primary outcome - result of CT CAP - was recorded as a binary variable (positive or negative).

Definition of a positive CT CAP

For the purposes of this study, a CT CAP was classified as positive if it demonstrated radiological evidence of a new extracranial lesion considered, on formal radiology reporting, to be malignant or suspicious for malignancy (i.e. a probable or confirmed primary or metastatic malignant process outside the central nervous system). Scans showing incidental, indeterminate, or benign findings without a radiological impression of malignancy were classified as negative. Where scan reports expressed diagnostic uncertainty, the categorisation applied by the reporting radiologist and/or the outcome of subsequent MDT discussion was used to assign a binary result.

Statistical analysis 

The outcome was the CT CAP result, which was a binary measure, either positive or negative. Due to the nature of the outcome, associations between patient factors and this outcome were assessed using logistic regression. The analysis was performed in two stages. Initially, the separate association between each factor and the CT CAP result was examined in a series of univariable analyses. Subsequently, the joint association between the patient factors and outcome was assessed in a multivariable analysis. In this analysis, the association between each factor and the outcome was adjusted for all other factors in the analysis. Only factors showing some evidence of an association with the outcome in the univariable analyses (p< 0.2) were included in the multivariable analysis.

Continuous variables were compared between conditions using analysis of variance (ANOVA) if found to be normally distributed, and using the Kruskal-Wallis test if not found to follow a normal distribution. Categorical variables were compared between conditions using Fisher’s exact test. Further analyses compared between patients who did and did not develop secondary resistance. Continuous variables that were normally distributed were compared between the two groups using the unpaired t-test, while continuous variables that were not normally distributed were analysed using the Mann-Whitney test.

## Results

One hundred and eighty-four patients were included; of these, 78 patients were excluded due to not having CT CAP completed, leaving 106 patients. Based on CT head findings and data collection, patients were categorised according to lesion location (supratentorial or infratentorial), number of lesions found on CT head, previous history of cancer and other comorbidities. A summary of the demographic characteristics of the patient cohort is reported in Table 1.

**Table 1:** Demographic/baseline characteristics Summary statistics are presented as mean ± standard deviation, or number (percentage).

Variable	n	Category	Summary
Age (years)	182	-	63.0 ± 25.7
Lesion location	169	Supratentorial	149 (88%)
		Infratentorial	20 (12%)
Number of lesions	162	-	1.5 ± 1.1
Number of lesions (categorised)	162	One lesion	123 (76%)
		Two lesions	23 (14%)
		Three or more lesions	16 (10%)
Lesion size	167	< 4 cm	57 (34%)
		≥ 4 cm	110 (66%)
Previous history of cancer	162	No	128 (79%)
		Yes	34 (21%)
Other comorbidity	162	No	45 (28%)
		Yes	117 (72%)

The mean age was 63.0 years (standard deviation (SD), 25.7; n = 182). Most patients had a supratentorial lesion, accounting for 149 of 169 patients with available lesion-location data (88%). The mean number of lesions was 1.5 (SD, 1.1; n = 162). Most patients had a single lesion, while 23 patients (14%) had two lesions and 16 patients (10%) had three or more lesions.

Lesion size was at least 4 cm in 110 of 167 patients (66%). A previous history of cancer was recorded in 34 of 162 patients (21%), while 117 patients (72%) had another recorded comorbidity.

CT chest, abdomen and pelvis outcomes

A definitive CT CAP outcome was available for 106 of the 184 patients included in the analysis. The CT CAP result was positive in 19 patients, corresponding to 18% of patients with an available outcome (95% confidence interval (CI), 11%-27%).

Univariable Analysis

On univariable analysis, lesion location was not significantly associated with a positive CT CAP result. A positive result was observed in 18 of 96 patients (19%) with a supratentorial lesion and in 1 of 10 patients (10%) with an infratentorial lesion.

There was a trend towards a higher likelihood of a positive CT CAP result with increasing lesion number. A positive result was observed in 9 of 96 patients (12%) with one lesion, compared with 24% of patients with two lesions and 50% of patients with three or more lesions. Relative to patients with one lesion, the odds ratio (OR) was 2.26 for patients with two lesions (95% CI, 0.60-8.44) and 7.33 for patients with three or more lesions (95% CI, 1.94-27.7). Overall, this association was of borderline statistical significance (P = 0.09).

Lesion size was also associated with a higher proportion of positive CT CAP results. A positive result was identified in 3 of 38 patients (8%) with lesions smaller than 4 cm, compared with 16 of 66 patients (24%) with lesions measuring at least 4 cm. Lesions measuring at least 4 cm were associated with higher odds of a positive result than smaller lesions (OR, 3.73; 95% CI, 1.01-13.8; P = 0.05).

The strongest univariable association was observed for a previous history of cancer. A positive CT CAP result was observed in 12 of 22 patients (55%) with a previous history of cancer, compared with 5 of 79 patients (6%) without a previous cancer history. The odds of a positive CT CAP result were 17.8 times higher in patients with a previous history of cancer (95% CI, 5.17-61.0; P < 0.001) (Table 2).

**Table 2:** Univariable associations with positive CT CAP result Evaluated via univariable logistic regression (likelihood ratio chi-squared). CT CAP: computed tomography of the chest, abdomen and pelvis; CI: confidence interval

Variable	Category	Positive CT CAP n/N (%)	Odds ratio (95% CI)	Test statistic	df	P-value
Lesion location	Supratentorial	18/96 (19)	1	χ² = 0.45	1	0.5
	Infratentorial	1/10 (10)	0.48 (0.06, 0.05)			
Number of lesions	One lesion	9/96 (12)	1	χ² = 4.83	2	0.09
	Two lesions	4/96 (24)	2.26 (0.60, 8.44)			
	Three or more lesions	6/12 (50)	7.33 (1.94, 27.7)			
Lesion size	< 4 cm	3/38 (8)	1	χ²=3.84	1	0.05
	≥ 4 cm	16/66 (24)	3.73 (1.01, 13.8)			
History of cancer	No	5/79 (6)	1	χ² = 23.11	1	<0.001
	Yes	12/22 (55)	17.8 (5.17, 61.0)			

Multivariable Analysis

Variables demonstrating evidence of an association with the outcome in the univariable analyses were entered into the multivariable logistic regression model. The model included the number of lesions, lesion size, and previous history of cancer.

After adjustment for lesion number and lesion size, previous history of cancer remained strongly associated with a positive CT CAP result. Patients with a previous history of cancer had substantially higher odds of a positive result than those without a previous cancer history (adjusted OR, 14.6; 95% CI, 4.09-52.4; P < 0.001).

Neither lesion number nor lesion size remained independently associated with the CT CAP outcome after adjustment. The adjusted OR was 1.22 for patients with two lesions and 3.05 for patients with three or more lesions, compared with patients with one lesion (overall P = 0.45). Lesions measuring at least 4 cm had an adjusted OR of 1.80 for a positive CT CAP result compared with lesions smaller than 4 cm (95% CI, 0.39-8.42; P = 0.45) (Table 3).

**Table 3:** Multivariable associations with a positive CT CAP result Evaluated via multivariable logistic regression (likelihood ratio chi-squared test). CT CAP: computed tomography of the chest, abdomen and pelvis; CI: confidence interval

Variable	Category	Odds ratio (95% CI)	Test statistic	df	P-value
Number of lesions	One lesion	1	χ² = 1.6	2	0.45
	Two lesions	1.22 (0.25, 5.83)			
	Three or more lesions	3.05 (0.54, 17.2)			
Lesion size	< 4 cm	1	χ² = 0.57	1	0.45
	≥ 4 cm	1.80 (0.39, 8.42)			
History of cancer	No	1	χ² = 18.23	1	<0.001
	Yes	14.6 (4.09, 52.4)			

## Discussion

Our results add to a small body of literature examining which clinical and radiological features of a newly identified brain lesion should prompt staging CT CAP. In this cohort, the overall positive yield of CT CAP was 18% (95% CI, 11-27%), meaning the majority of patients who underwent staging imaging did not have a systemic malignancy identified. This is broadly consistent with the findings of previous work, suggesting that CT CAP is a moderate-to-low yield investigation overall, and that its value is concentrated in specific subgroups rather than distributed evenly across all patients presenting with a new brain lesion. A comparable dedicated analysis of CT CAP in patients with solitary and multiple brain lesions similarly concluded that chest imaging was warranted in this population, but that abdominopelvic imaging added little in patients without other clinical or radiological features, suggesting an abdominopelvic primary. Taken together with our findings, this supports a move away from a "one CT CAP fits all" approach toward a more selective, risk-stratified strategy [10].

Previous history of cancer as the dominant predictor

The strongest and most consistent predictor of a positive CT CAP in our cohort was a previous history of cancer, with an unadjusted OR of 17.8 (95% CI, 5.17-61.0) that remained essentially unchanged after adjustment for lesion number and size (adjusted OR, 14.6; 95% CI, 4.09-52.4). This aligns closely with the existing literature on the radiological approach to brain masses. Reviews of brain metastasis diagnosis emphasise that in patients with a symptomatic intracranial lesion and a known diagnosis of an extracranial primary cancer, imaging findings are highly suggestive of metastasis, whereas in patients without a cancer history, a solitary brain mass is more likely to represent a primary brain tumour. Our data provide a direct, quantified illustration of this principle in the context of staging decision-making: a known cancer history essentially identifies the subgroup in which CT CAP is genuinely diagnostic, while its yield in patients without such a history was low (6%). This is consistent with case-based literature describing brain lesions discovered incidentally during staging or surveillance of a known primary tumour, where the presence of a previous malignancy substantially raises pre-test probability of a metastatic aetiology [11].

Number and size of lesions

Multiplicity of lesions showed a graded, borderline-significant association with a positive CT CAP on univariable analysis (12% for one lesion, 24% for two lesions, 50% for three or more; P = 0.09), but this association was substantially attenuated after adjustment for cancer history and lesion size (adjusted OR 1.22 and 3.05, respectively; P = 0.45). This pattern mirrors the broader neuro-oncological literature, which generally treats multifocality as a radiological feature suggestive of, rather than independently diagnostic for, metastatic disease. Authoritative reviews note that the presence of multiple lesions, especially spanning multiple intracranial compartments, is effectively diagnostic of metastasis, and neurology literature similarly states that contrast-enhanced imaging findings of multiple lesions are strongly suggestive of metastases, particularly in a patient with a known diagnosis of cancer. Our multivariable findings suggest that much of the apparent predictive value of lesion number in univariable analysis may be explained by its correlation with an underlying cancer history rather than representing an independent driver of systemic staging yield - an important distinction for pre-test probability models, because it implies that lesion count alone should not be used to justify CT CAP in patients without other risk features [12].

These findings support a targeted rather than universal approach to CT CAP in patients with a newly diagnosed brain lesion. A previous history of cancer identifies a high-yield subgroup in which staging is clearly justified, consistent with the broader principle in oncological imaging that a known malignancy dramatically raises the pre-test probability of a positive staging scan and that basic staging investigations, when appropriately targeted, carry good diagnostic yield. This mirrors findings in the metastatic malignancy of unknown primary literature, where a basic diagnostic panel - including cross-sectional imaging alongside history, examination, and biopsy - accounted for the substantial majority of primary tumours eventually identified. Conversely, in patients without a cancer history, our results suggest CT CAP yield is low even when multiple or large lesions are present, raising the question of whether resources, radiation exposure, and incidental-finding burden could be reduced by reserving CAP staging for patients with a known cancer history or other specific red-flag features, rather than applying it uniformly. This is consistent with observations in other tumour staging contexts that low pre-test probability imaging carries a meaningful false-referral burden without proportionate diagnostic benefit [13].

Limitations

Several limitations should be considered when interpreting these results. First, this was a retrospective single-centre analysis with substantial missing data: only 106 of 184 originally identified patients had a definitive CT CAP outcome available, and the denominators for individual variables in Tables 1-3 varied further (e.g. lesion location data were available in only 169/184, and complete case multivariable analysis was based on a smaller subset still), raising the possibility of selection or missing-data bias if incomplete workup was non-random with respect to lesion characteristics. Secondly, we did not have granular data on specific ancillary radiological findings (e.g. pulmonary nodules, lymphadenopathy) known to influence CAP yield independent of lesion count or size, nor on other clinically relevant staging predictors such as presenting symptoms, performance status, or laboratory tumour markers. Finally, as with any retrospective study, causal inference is limited, and prospective validation of a risk-stratified staging pathway (for example, incorporating cancer history as the primary trigger for CT CAP) would strengthen the case for practice change.

## Conclusions

A previous history of malignancy was the sole independent predictor of a positive staging CT CAP in patients with an NBL, with an adjusted OR of 14.6. Lesion number, size, and location did not retain significance after adjustment, suggesting these radiological features carry limited discriminatory value for predicting extracranial malignancy once oncological history is accounted for, challenging their reflexive use as triggers for staging imaging. These findings support a history-driven, risk-stratified approach to CT CAP. Patients without a known cancer history appear to derive low diagnostic yield from routine staging, regardless of how radiologically suspicious a lesion appears (e.g. multiple lesions, ring-enhancement). If validated, prioritising cancer history over lesion morphology could reduce unnecessary radiation exposure, contrast-related risk, incidental findings requiring further work-up, cost, and delay to neurosurgical intervention in patients without a known primary malignancy. Given the retrospective, single-centre design and wide CIs around key estimates, prospective multicentre validation is warranted, with standardised CT CAP protocols and reporting criteria, adequately powered subgroup analysis by cancer type and remission latency, and formal cost-effectiveness modelling against universal staging. Ultimately, a stepped-wedge or pragmatic trial evaluating a revised staging algorithm (staging CT CAP guided primarily by cancer history rather than lesion characteristics) against current standard practice would provide the strongest evidence for future studies to build on and potential evidence for a change in clinical guidelines.
